# Update on sex-specific microglia contribution to early synaptic dysfunction in Alzheimer’s disease

**DOI:** 10.3389/fnmol.2026.1905780

**Published:** 2026-08-24

**Authors:** Candice M. Roux, Slavica Krantic

**Affiliations:** 1 Department of Cell and Developmental Biology, Division of Biosciences, University College London, London, UK; 2 Neuroimmunology, Inflammation and Therapeutics Laboratory, Inserm U938, Centre De Recherche Saint-Antoine (CRSA), Sorbonne Université, Paris, France

**Keywords:** glutamate neurotransmission, neuron-microglia crosstalk, sexual dimorphism, synaptic plasticity, transcriptomic and metabolic microglia profiles

## Abstract

Women are twice as likely to develop Alzheimer’s disease (AD) as men. Despite substantial progress in understanding the pathogenesis of sporadic AD, sex-specific mechanisms remain insufficiently explored, and preclinical research has historically been biased toward male subjects. Furthermore, there is often a delay of more than 10 years between the onset of neuropathological changes and clinical diagnosis. This latent pre-symptomatic period, during which pathological alterations may still be reversible, represents a promising window for therapeutic intervention. In this context, synaptic dysfunction and neuroinflammatory alterations are among the earliest detectable AD-associated impairments and have recently emerged as promising therapeutic targets. This mini-review summarizes the currently limited knowledge on sex-related differences in synaptic functions during pre-symptomatic stage of AD pathology in both pre-clinical and clinical studies. We further position sex as a critical biological variable impacting neuronal activity, either directly or indirectly through microglia-neuron crosstalk. Finally, we emphasize the importance and rationale for integrating sex-specific neuroimmune mechanisms into early-stage research to guide design of targeted, sex-tailored therapeutic strategies that modulate neuron-microglia interactions before clinical onset.

## 1 Introduction

Alzheimer’s disease (AD) is incurable neurodegenerative disease and among the major global health problems. There is a marked sexual dimorphism of AD, with nearly two-thirds of patients being women. In addition to their higher prevalence in AD, women exhibit more severe clinical symptoms and faster rate of disease progression (Podcasy and Epperson, 2016), as well as higher tau protein levels (Edwards et al., 2021) and more Aβ pathology (Oveisgharan et al., 2018) compared to men. Of utmost importance, clinical trials suggest a differential response to AD therapies, including anti-amyloid therapies, with women experiencing less benefits than men (for review Lynch, 2024). Despite these observations, mechanisms underlying such differences have been understudied. Although animal models have provided substantial insight into AD pathogenesis, preclinical research has historically relied predominantly on male subjects. Nevertheless, recent years have seen increasing efforts to include female animals and directly investigate sex-dependent mechanisms underlying AD pathology and progression (Lecordier et al., 2022; Pluta, 2025; Pluta and Ułamek-Kozioł, 2026; Rodrigues et al., 2025).

Importantly, AD is characterized by a long pre-symptomatic period (10–20 years), during which neuropathological changes accumulate silently up to clinically detectable symptoms. This latent stage has become a major focus of research since it corresponds with the limited time-window while initial dysfunctions are reversible back to physiological state, thereby maximizing the efficacy of disease-modifying treatments (Riedel et al., 2016). In line with this perspective, recent framework has been established for early detection of neuropathologic changes in asymptomatic AD, recognizing the need for identification of reversible targets. Among the earliest detectable alterations, synaptic dysfunction and neuroinflammatory changes have emerged as key contributors to AD progression. Microglia-neuron interactions are increasingly recognized as central regulators of synaptic homeostasis and early neuronal dysfunction in AD, positioning microglia as promising therapeutic targets. Therefore, a better understanding of sex-specific differences in microglia-neuron interaction during the pre-symptomatic stage of AD may help refine the development of sex-tailored therapeutic strategies.

This mini-review summarizes current preclinical and clinical evidence on sex-related differences in brain function and vulnerability to AD. We focus on how biological sex shapes neuroimmune responses by emphasizing sex-dependent early synaptic dysfunction and properties of microglia. We further discuss how these two cell types functionally converge to influence AD pathogenesis, highlighting both direct and indirect pathways behind sex-related impact on neurodegeneration.

## 2 Impact of sex on neuronal (dys)function

Aβ and hyperphosphorylated tau protein (p-tau) exert well-established synaptotoxic effects and are major contributors to early AD-associated synaptic dysfunction. According to the amyloid hypothesis, these pathological alterations are associated with a progressive shift toward neuronal hyperexcitability, characterized by excessive glutamatergic signaling and reduced inhibitory GABAergic neurotransmission (Palop and Mucke, 2010). Using open access series of imaging studies (*n* = 437 studies), this concept was further extended as it showed evidence that cerebral excitation/inhibition (E/I) balance displays sexual dimorphism and that its disruption in the hippocampus correlates specifically to pre-symptomatic AD stage only in female patients (Fortel et al., 2023).

In the following section, we will first provide a brief overview of the sparse literature on sex-specific GABAergic changes before focusing more extensively on excitatory glutamatergic signaling in the context of AD. The relatively limited attention given to GABAergic neurotransmission is historical and related to the longstanding view that GABAergic neurons are more resilient to aging and less vulnerable to AD than other types of neurons, including glutamatergic ones (Rissman and Mobley, 2011). However, recent data support that AD is associated with alterations throughout the GABAergic system, including expression levels of GABA, glutamate decarboxylase (GAD 65/67) and GABA receptors (GABA_*A*_ and GABA_*B*_), as well as GABA transporters (GAT 1–3) and vesicular GABA transporter VGAT.

### 2.1 Sex differences in GABA signaling

Interestingly, during brain development, the GABAergic system represents one of the endpoints of sexual differentiation, ultimately leading to sex-specific regulation of GABAergic signaling (e.g., somatostatin release) and differences in GABA receptor subunit composition and function. However, relatively little is known about sex-specific alterations of the GABAergic system in AD, and the available evidence remains limited and sometimes inconsistent. For example, using nuclear magnetic resonance in Tg2576 mice, females demonstrated higher GABA content in the hippocampus as compared to males (Roy et al., 2018). In contrast, untargeted mass spectrometry in 3xTg model did not evidence any difference in the whole brain GABA content between sexes (Strefeler et al., 2023). These discrepancies likely reflect differences in disease model, brain region analysed, disease stage and analytical methodology, highlighting the need for region- and cell-specific investigations. Indeed, 17β-estradiol (E2) acutely suppresses GABAergic inhibition in the hippocampus of female rats through a sex-specific oestrogen receptor α (ERα), mGluR, and endocannabinoid-dependent mechanism (Tabatadze et al., 2015). These findings suggest that males and females may exhibit similar baseline inhibitory transmission while relying on distinct molecular mechanisms to regulate inhibitory synaptic plasticity, raising the possibility that AD pathology could differentially disrupt these sex-specific regulatory pathways. Besides, APP knock-in APP*^NL–G–F^* mice display higher α1 and α5 GABA_*A*_ receptor subunits expression in male than female hippocampus suggesting sex-dependent regulation of inhibitory neurotransmission during AD progression (Auta et al., 2022).

Human studies of GABAergic system in AD have either not assessed sexual dimorphism or reported contradictory data. For instance, a recent systematic meta-analysis indicated AD-associated decrease in expression of brain GABA, GABA_*A*_ receptors, GAD65/67 and GABA transporters but did not assess a putative sex impact (Carello-Collar et al., 2023). Another systematic review of transcriptomic studies revealed a more significant impairment in expression of cortical genes related to regulation and organization of GABA (and glutamate) neurotransmitter pathways in female than in male AD patients (Lopez-Lee et al., 2024). However, independent study using western blot to assess the level of relevant proteins’ expression found no difference in either GABA_*A*_ receptors subunits or GAD65/67 expressions in the entorhinal cortex and hippocampus of AD patients (Ethiraj et al., 2021). The latter study additionally reported a complex correlation (negative or positive) with age, which was both sex- and brain region (cortex versus hippocampus) dependent (Ethiraj et al., 2021). These discrepancies highlight the high dependency of these differences on the brain region, age and receptor sub-type considered.

Overall, the current literature does not provide consistent or conclusive evidence regarding sexual dimorphism of GABAergic dysfunctions at overt stages of AD pathology whereas its relevance along pre-symptomatic stage remains unexplored. Many reasons could explain these discrepancies such as insufficient standardization of disease staging, experimental protocols and procedures as well as analyses methodology. Future studies should address these limitations by implementing harmonized experimental designs and region- and cell type-specific analyses, thereby enabling more reliable comparisons and facilitating the integration of findings across studies.

### 2.2 Sex differences in basal glutamate signaling

Rodents exhibit sex-specific brain-region differences in glutamate levels and receptors (i.e., mGLURs, NMDA, AMPA, kainate) distribution and density. Under healthy conditions, females show greater type 2, 3 and 5 metabotropic glutamate receptor (mGLURs) expression in the hippocampus, than males. Expression of the NR1 and NR2B subunits of NMDA receptor in the hippocampus is also higher in female than male rats (Wang et al., 2015). Some of sex-specific differences can be explained by interactions between sex hormones and glutamatergic transmission. For instance, oestrogens modulate expression and function of glutamate receptors, as well as astrocytic glutamate uptake across the oestrous cycle. Variations in NMDA, AMPA and kainate receptor densities have also been shown to be overall reduced in oestrus females as compared to male rats (Palomero-Gallagher et al., 2003). Remarkably, 17β-oestradiol (E2) ameliorates Aβ-induced deficits in synaptic plasticity in neuronal cultures, as well as *in vivo* in ovariectomized rats, via GluR1-containing AMPA receptor (Logan et al., 2011). In agreement, E2 modulates LTP in the hippocampus (Copenhaver and LeGates, 2024; Foy et al., 1999; Sawai et al., 2002). Moreover, glutamate transport across neuronal membranes is also influenced by oestrous cycle. For instance, in rat cortical synaptosomes, the affinity of glutamate for its high-affinity transporter is significantly greater in proestrus and oestrus females than in males and diestrus females (Mitrovic et al., 1999).

As for human studies, one revealed that blood glutamate levels are significantly higher in men than in women and vary across the menstrual cycle with glutamate levels showing an inverse relationship with blood oestrogen and progesterone levels (Zlotnik et al., 2011).

### 2.3 Sex-dependent vulnerability of glutamatergic neurons in early AD

Direct interaction between Aβ oligomers and glutamate receptors yields increased inward currents and intracellular calcium via AMPA and NMDA receptors (Alberdi et al., 2010), hence creating an environment for increased neuronal susceptibility to excitotoxicity (Nong et al., 2025). In addition, soluble Aβ binds to vesicular glutamate transporter-1 (VGLUT1), potentiating glutamate release.

In an AD rat model McGill-R-Thy1-APP, a decrease in glutamate levels within the dorsal hippocampus was reported in male but not in female, highlighting sex-dependent alterations in glutamatergic homeostasis (Nilsen et al., 2014). Interestingly, in APP^*NL–F*/*NL–F*^ mice, female display more efficient maintenance of glutamate homeostatis than male, with no age-related decrease in evoked glutamate release in entorhinal cortex and hippocampus. Moreover, young females showed better glutamate clearance as compared to age-matched wild-type (WT) controls suggesting that oestrogen may play a protecting role against glutamate hyperexcitability at early stages, likely by supporting astrocyte function and inhibitory regulations (Soni et al., 2024).

Among rare human studies, one has reported that post-mortem female AD patients display lower expression of Glu2-containing AMPA receptors in cholinergic Nucleus Basalis neurons (Counts et al., 2011). Hence, it appears that oestrogen can exert neuroprotective effects under physiological conditions. Oestrogen declines with aging and menopause may exacerbate glutamatergic dysregulation thereby increasing susceptibility to synaptic dysfunction and neuronal loss.

Increasing interest in the study of sex-specific differences on neuronal activity in AD has emerged during the recent years in both rodents and humans (Dennison et al., 2021; Lecordier et al., 2022; Pluta, 2025; Pluta and Ułamek-Kozioł, 2026; Rodrigues et al., 2025), as best illustrated in the 3xTg model (Dennison et al., 2021). In APP/PS1d9 model, females aged 4–5 months display faster long-term potentiation (LTP) decay and stronger associative memory impairment compared to their WT littermates than males (Navakkode et al., 2021). Another study in APPPS1 model, reported that basal synaptic activity, presynaptic release probability and synaptic plasticity including LTP and long-term depression (LTD), are significantly impacted by stage of AD pathology, but appear independent of sex (Sha et al., 2023). Remarkably, investigating glutamatergic neurotransmission more in details by refining electrophysiological analyses, the same group reported opposite alterations in hippocampal CA1 neurons’ basal neurotransmission and response to excitatory inputs between male and female APPPS1 mice. Indeed, weakening of synaptic efficacy in APPPS1 as compared to control mice was more pronounced in females than in males. Of utmost importance, these complex and subtle alterations of neuronal activity preceded plasticity impairments as they did not translate yet into sexual dimorphism of LTP and LTD at the pre-symptomatic stage (Chaigneau et al., 2025). However, these findings need to be confirmed by independent studies before conclusions can be drawn. Consistent with this notion, in a non-transgenic mouse model of AD (intracerebroventricular injection of Aβ_1–42_), impairments in both short- and long-term synaptic plasticity were reported to be independent of sex (Jiménez-Herrera et al., 2023). Together with findings from APPPS1 mice, these observations suggest that sexual dimorphism in neuronal activity may emerge preferentially during specific stages of pathology or may only become apparent when more sensitive electrophysiological parameters are examined. Therefore, while a growing body of evidence supports increased female vulnerability to AD-associated synaptic dysfunction, the magnitude and direction of sex effects likely depend on the mechanisms initiating pathology and the temporal stage at which neuronal function is assessed. Such variability suggests that neuronal dysfunction may not solely reflect direct effects of amyloid or tau pathology but may also involve sex-dependent regulation by neighboring glial cells, particularly microglia. Future studies should therefore consider neuron-microglia interactions to provide a more integrated view of sexual dimorphism of neuronal (dys)functions in general, particularly with respect to glutamatergic signaling.

Studies in humans are much more limited, particularly at the pre-symptomatic stage, since synaptic alterations are difficult to assess ante-mortem. Potential direct evidence of neuronal dysfunction stem from epidemiological data, suggesting a transient hyperexcitability as manifested by early-stage AD epileptiform activity (Amatniek et al., 2006; Gleichmann et al., 2011). However, male versus female differences have not been investigated so far. Studies aimed on exploring sex impact on neuronal excitability are greatly warranted in future clinical and epidemiological settings.

## 3 Sexual dimorphism in microglia

Microglia are the resident immune cells of the brain. They continuously monitor neuronal health and engage in complex bidirectional communication with neurons, which is essential for brain development, maintenance of homeostasis and resolution of neuroinflammation (Wang et al., 2026). Increasing evidence point to sexual differences in microglia both in health and disease (for review Lopez-Lee et al., 2024).

Transcriptome sequencing demonstrated sexual dimorphism in adult murine microglia with female microglia having a neuroprotective phenotype, independent from hormonal cues, which is retained after transfer into male brains. Furthermore, transplantation of female microglia protects male brains from acute neuroinflammation and injury (Villa et al., 2018).

In human, by parturition, there are significant sex differences in the number and morphology of microglia in almost every brain region including the hippocampus, parietal cortex, and amygdala (Schwarz et al., 2012). Females display more microglia than men in part due to gonadal steroids impact during perinatal period. The control of neuronal activity by microglia is also suggested to be sex-dependent and different between brain regions (Barko et al., 2022; Zhang et al., 2025). Thus, in the preoptic area, males have more microglia with an “activated” morphology characterized by an increase in cell body size and a decrease in process length and branching (Lenz et al., 2013).

In the context of neurodegenerative diseases, compelling evidence suggests that AD may be considered as a disorder of neuroimmune regulation (Aranda-Abreu et al., 2026). However, the precise role of microglia in driving AD pathology remains a matter of debate with both beneficial and deleterious effects of microglia activation being reported. Such controversy likely reflects spatial and temporal (early pre-symptomatic *versus* late, symptomatic stages) complexities (Roveta et al., 2024).

### 3.1 Sex-specific transcriptional signatures of microglia

Increased microgliosis has been associated with alterations in homeostatic (M0) and Disease-Associated Microglia (DAM)/microglial neurodegenerative phenotype (MnDG) genes expressions in various AD mouse models (Kang et al., 2018; Keren-Shaul et al., 2017; Krasemann et al., 2017; Stephen et al., 2019): These studies demonstrated AD-related decrease of selected M0 genes expression (e.g., *Tmem119*, *P2ry12* and *Cx3cr1…*) as well as the central role of APOE-TREM2 (Triggering Receptor Expressed on Myeloid cells 2) pathway in triggering increase in DAM genes expression (e.g., *Clec7a*, *Lpl*, *Lgals3*, *Apoe*, and *Spp1…*). This highlights the pivotal role of TREM2 in the switch from M0 to DAM phenotype (Krasemann et al., 2017). Sex-differences in M0/DAM phenotypes remain poorly addressed. Only a very recent study in pre-symptomatic APPPS1 mice reported increased microgliosis which translated into larger microglia coverage in male than female hippocampus and was associated with sex-related differences in expression of either homeostatic M0 or DAM markers, with the latter being much more affected. Specifically, *Spp1* and *Clec7a* expressions were higher in female than in male mice at the studied pre-symptomatic stage (Chaigneau et al., 2025). However, as these findings originate from a single study, they require confirmation in independent cohorts and AD models. In another study, TREM2-dependent interaction between plaques and microglia has been reported to be sex-independent at least in 5XFAD mice with APOE4 knock-in even if this effect strongly relied on APOE genotype. Indeed, 5XFAD mice with APOE3 knock-in displayed reduced plaque size and microglia plaque coverage only in female mice (Stephen et al., 2019). However, these findings also need to be confirmed by additional, independent studies.

Using a different mouse model, researchers identified a distinct population of microglia: activated response microglia (ARMs), characterized by expression of a specific set of genes. This study reported in addition that the switch from homeostatic M0- toward the ARMs signature occurred more rapidly in female APP*^NL–G–F^* mice as compared with males (Sala Frigerio et al., 2019). A more recent study reported increased expression ARMs/DAMs genes in mouse APP/PS1dE9 female microglia compared to male (Guillot-Sestier et al., 2021).

In humans, a sex-specific TREM2 and TLR7 dysregulation was observed in AD patients’ brains in comparison to relevant controls. Such dysregulation was more pronounced in women than men, at least in the temporal cortex (Bonham et al., 2019). An independent study reported transcriptomic up-regulation of microglial pro-inflammatory and immune activation-related genes, including APOE and TLR, which were enriched in women post-mortem AD brain samples as compared to men (Mathys et al., 2019).

Recently, a very thorough study combining analysis of seven transgenic AD mouse models and human AD cohorts identified a highly conserved transcriptomic module encompassing TYROBP, TREM2 and complement C1Q component. This signature is specifically expressed by a particular subpopulation of microglia involved in synaptic pruning, immune activation and control of synaptic excitability (Li et al., 2026). However, sex dimension of these mechanisms has not been assessed yet but this opens interesting perspectives.

### 3.2 Sex-specific metabolic signatures of microglia

Alterations in metabolism are detectable at early stages of AD, and impaired energy metabolism precedes cognitive decline, suggesting that mitochondrial dysfunction may be a causal factor in the pathogenesis (Ferretti et al., 2018). This has been shown to be tightly related to homeostatic microglia shifting from oxidative phosphorylation toward glycolysis in neuroinflammatory conditions (Mi et al., 2021).

Interestingly, mitochondrial functions in both microglia and neurons are influenced by estrogens (Irwin et al., 2012; Klinge, 2017). In 3xTg mouse model, ovariectomy resulted in decreased mitochondrial respiration and increased mitochondrial Aβ levels, the latter being reversed with estrogen treatment (Zhao et al., 2023). These results illustrate the protective role of estrogen on mitochondrial function. Remarkably, such estrogen effect was correlated with mitigating cognitive decline seen in ovariectomized 3xTg mice, as well as with decreased amyloid and tau protein pathologies, increased mitophagy and mitochondrial biogenesis (Zhao et al., 2023). In addition, in APP/PS1dE9 mice, only female but not male microglia shift to glycolysis in the presence of amyloid plaques (Guillot-Sestier et al., 2021).

In line with animal models, ante-menopausal women display heightened levels of antioxidant enzymes and lower levels of hydrogen peroxide, NADPH oxidase and homocysteine than men. This points to a beneficial impact of female sex hormones on metabolic processes and mitochondrial function (Demarest and McCarthy, 2015; for review see Lopez-Lee et al., 2024; Tenkorang et al., 2018). Decrease in estrogen levels after menopause alleviates this sex difference, reducing antioxidant capacity and increasing reactive oxygen species (ROS) production to levels similar to those in men (Demarest and McCarthy, 2015).

## 4 Microglia-neuron crosstalk in early pathology: the impact of sex

Chronic inflammatory state caused by Aβ accumulation and subsequent microgliosis can trigger the release of excessive pro-inflammatory cytokines and neurotoxic molecules. This creates a hostile microenvironment that damages neurons and ultimately exacerbates neurodegeneration. However, the impact of sex in these mechanisms remains contradictory.

For instance, equal amyloid pathology in males and females at pre-symptomatic stage in APPPS1 mouse correlates with lack of sex impact on electrophysiological parameters (Sha et al., 2023). By contrast, in spite of equivalent Aβ pathology in male and female TgCRND8 mice along the pathogenesis, females display cognitive deficits earlier than males (Granger et al., 2016). Other studies reported that earlier amyloidosis in females, translates into more impaired neuronal activity at overt stage of AD-like pathology in APP/PS1d9 model (Jiao et al., 2016). Interestingly, using a similar model to study early pre-symptomatic stage, a recent study reported that, despite higher Aβ load, females display fewer amyloid plaques and more efficient synaptic transmission than males. Hence, in this model, more resilient synaptic transmission along with less microgliosis point to better early coping with pathogenesis in females than in males (Chaigneau et al., 2025). Although this intriguing hypothesis requires validation in independent studies, it raises the possibility that females initially display greater resilience before becoming more vulnerable at later stages of disease progression. Future studies should test this hypothesis using longitudinal *in vivo* electrophysiological recordings combined with behavioral assessments and neuropathological imaging including multi micro-PET imaging of Aβ, p-tau protein and inflammatory microglia (Kang et al., 2022) to better characterize the temporal evolution of sex-specific neuronal dysfunction

Another study using the ecologically relevant IntelliCage paradigm reported that, independent of Aβ burden, aged APP/PS1 females exhibited superior cognitive performance compared to males (Mifflin et al., 2021). Similar results were further obtained by testing the same set of animals in a classic Morris Water Maze task. Data from this study are thus in contrast with reported greater cognitive impairment in APP/PS1d9 females than males (for review Jankowsky and Zheng, 2017). Such discrepancy supports a complex interplay between Aβ pathology and cognitive symptoms, thus indicating that the impact of pathology on neuronal function may also differ according to AD-like models. As a corollary, previously reported sex differences in synaptic activity may be related to different kinetics of amyloidosis between males and females, depending on AD model. Interestingly, this is contrasting with reported alterations in synaptic activity which appear as a general feature across several AD-like mice models (see Mango et al., 2019 for review). Rather than representing contradictory findings, the apparent discrepancies across AD models likely reflect differences in the pathological context captured by each experimental paradigm. Indeed, transgenic and knock-in models differ substantially in their genetic background, the kinetics and extent of amyloid deposition, the presence or absence of tau pathology, and the temporal progression of synaptic dysfunction. Consequently, sex-dependent phenotypes may emerge at different stages of disease progression depending on the underlying pathological mechanisms. In addition, differences in the brain regions investigated, the neuronal circuits analysed and the electrophysiological or behavioral endpoints selected may further contribute to the observed variability. Therefore, the current literature may capture different stages and facets of sex-dependent AD pathophysiology rather than truly conflicting biological observations. Within this framework, future studies are needed to decipher the underlying mechanisms of synaptic dysfunctions, especially in models where they appear independent of amyloidosis. Hence, assessing microglia and their associated neuroinflammatory activity would be of highest interest. In this light, a cytokine TNFα, known both as a major mediator of (neuro)-immune interactions and a key regulator of synaptic excitability (Olmos and Lladó, 2014), is of particular interest. Indeed, Aβ has been reported to trigger TNFα production by microglia (Meda et al., 1995). Accordingly, Chaigneau et al recently reported that expression of *tnf*α and type-2 receptor of TNFα (*tnfr2*) correlate with Aβ load in pre-symptomatic male APPPS1 mice (Chaigneau et al., 2025). Although the data on *tnf*α/*tnfr2* come from a single study and would have to be confirmed in additional studies, they raise the hypothesis that TNFα may exert a beneficial role during the earliest stages of AD pathogenesis (Steeland et al., 2018). Besides, in contrast to type-1 receptor (*tnfr1*) (He et al., 2007)*, tnfr2* has been demonstrated to play a protective role in AD-like amyloidosis and microgliosis (Jiang et al., 2014), further aligning with higher expression of cortical *tnfr2* and lower amyloid plaques coverage in female APPPS1 hippocampus (Chaigneau et al., 2025). Together, these observations are consistent with the hypothesis that TNFα signaling, particularly through TNFR2, may contribute to adaptive neuroimmune responses during the earliest stages of AD pathogenesis (Steeland et al., 2018).

From a translational perspective, Rueda-Carrasco et al demonstrated that microglia engulf hyperactive synapses *via* TREM2 yielding resolution of neuronal hyperactivity using hAPP NL−F knock−in mouse model which recapitulates Aβ oligomers-induced synaptic hyperactivity characteristic of earliest AD stages. Furthermore, TREM2 loss−of−function results in disruption of microglial removal of hyperactive synapses and sustained neuronal hyperactivity (Rueda-Carrasco et al., 2023). This elegant study, though not specifically assessing the sex dimension of the studied parameters, has explicitly demonstrated that appropriate interventional strategies targeting microglia could ameliorate neuronal function by exploiting interplay between Aβ accumulation, microglia activation and neuronal function.

## 5 Conclusion

**FIGURE 1:**
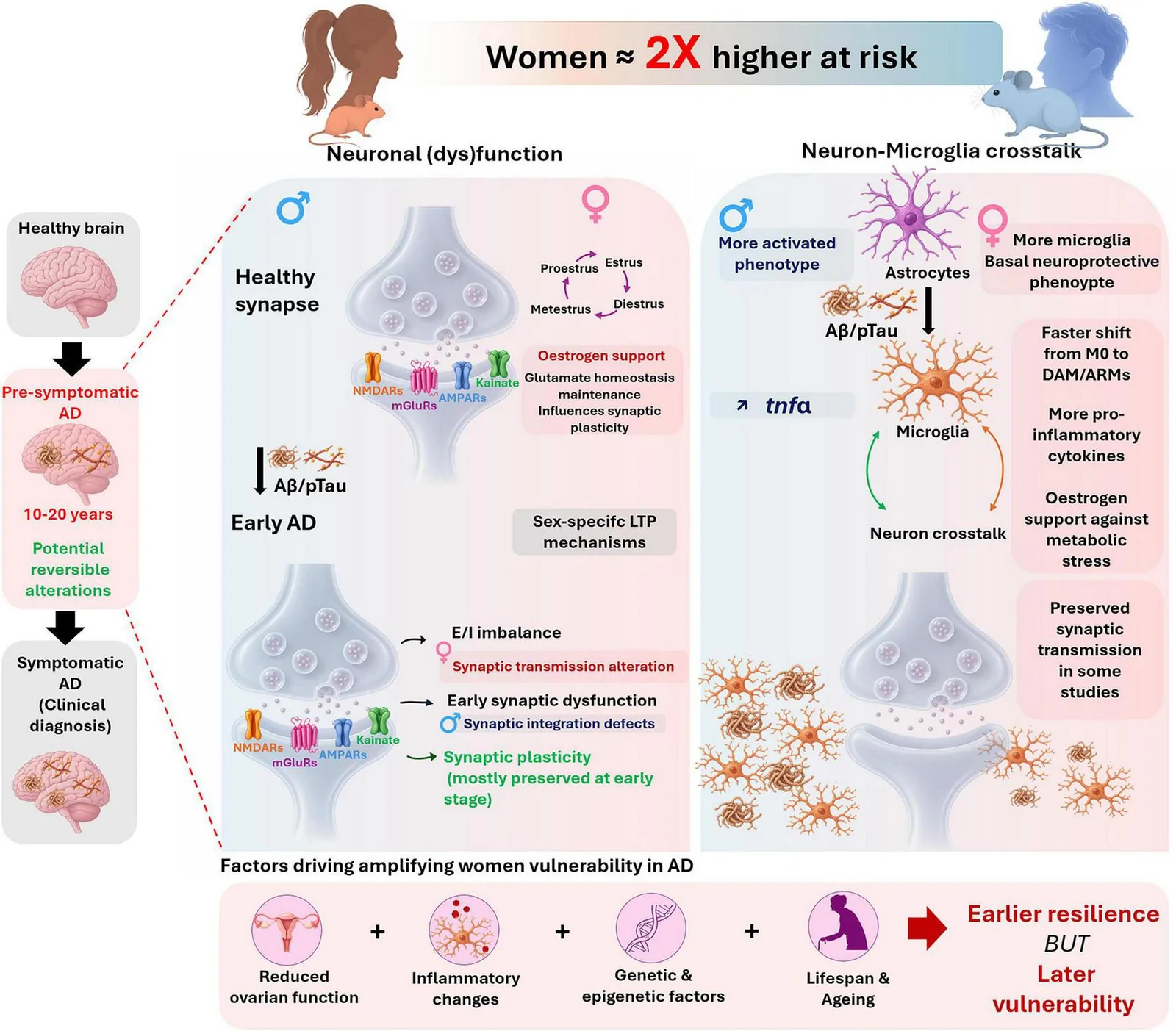
Proposed framework of sex-specific neuronal dysfunction and neuron–microglia crosstalk during the early stages of Alzheimer’s disease. Top and left panels: Women are approximately twice as likely as men to develop Alzheimer’s disease (AD). This increased risk has been consistently reported in both clinical studies (illustrated by the human silhouettes) and preclinical models (illustrated by the mouse icons). Most of the preclinical data stem from male studies. During the long pre-symptomatic phase of AD (approximately 10–20 years before clinical diagnosis), soluble Aβ and tau species induce early alterations in neuronal and glial function that may remain partially reversible. Left panel: Sex-dependent neuronal (dys)function. In the healthy brain, oestrogen contributes to the maintenance of glutamatergic homeostasis and synaptic plasticity. During early AD, emerging evidence suggests sex-dependent alterations in excitatory neurotransmission, including changes in excitation/inhibition (E/I) balance, synaptic transmission and neuronal integration, whereas long-term potentiation (LTP) and long-term depression (LTD) are often preserved at pre-symptomatic stages. Right panel: Sex-dependent neuron–microglia crosstalk. Microglia display sexually dimorphic phenotypes under physiological conditions. During early AD, neuronal dysfunction and microglial activation interact bidirectionally, potentially leading to sex-specific inflammatory responses. Available evidence suggests that female microglia initially exhibit a more homeostatic/neuroprotective phenotype, whereas disease progression may promote a faster transition toward disease-associated microglia (DAM) or activated response microglia (ARM), accompanied by increased production of pro-inflammatory mediators. Conversely, male microglia may exhibit a more activated phenotype during early disease stages. Bottom panel: The combined influence of ovarian ageing, inflammatory changes, genetic and epigenetic factors, and lifespan-related processes may contribute to the greater lifetime susceptibility of women to AD. Based on the currently available evidence, we propose the hypothesis that females may display greater resilience during the earliest stages of AD, followed by increased vulnerability as pathology progresses.

This review highlights crucial sexual dimorphism in neurons and microglia physiology and their interaction under healthy conditions that could to some extent explain the differences observed in coping with AD pathogenesis. Sexual dimorphism in neuron-microglia interactions emerge as a critical factor shaping AD vulnerability and progression. Evidence from preclinical models suggests that females exhibit a distinct basal neurobiological state, characterized by more efficient glutamatergic homeostasis, enhanced synaptic plasticity, and a more tightly regulated microglial profile which may initially confer resilience to early AD-related insults. However, the decline in ovarian hormones with ageing appears to disrupt this balance, perhaps increasing susceptibility to excitotoxicity, neuroinflammation, and subsequent neurodegeneration (Figure 1).

Despite growing recognition of these differences, major gaps remain, largely due to the historical underrepresentation or inadequate reporting of sex in experimental studies [*i.e.*, in more than 20% of the published studies on AD, the sex of the animals used has even not been indicated (Dennison et al., 2021)]. To overcome current limitations, future preclinical studies should not only incorporate sex as a biological variable but also account for heterogeneity of animal models such as genetic background in transgenic paradigms, as well as the use of models recapitulating either isolated (amyloid or tau protein) or combined proteinopathies. Furthermore, future studies should characterize the temporal evolution of amyloid and tau pathology in a sex-specific manner and favor region- and cell type-specific analyses over whole-brain approaches, as the latter may obscure biologically relevant differences through compensatory changes across brain regions or cell populations. Such comparative approaches will be essential to determine whether the reported discrepancies reflect genuine biological differences between models or distinct temporal windows of the same pathological process. Finally, neuronal and microglial dysfunctions should not be assessed independently but rather in an integrated context of neuron-microglia crosstalk. For instance, microglia express a variety of neurotransmitter receptors and initial loss of these receptors due to neuronal loss could be followed by their increased expression at later stages of AD pathogenesis due to microgliosis, as recently proposed for metabotropic mGlu5 receptors (Wang et al., 2025). Altogether, the implementation of more integrated and standardized models and procedures should help overcome some of the limitations that have hindered translation to clinical settings, where sex-specific responses to AD therapies are increasingly evident. The dynamic shift observed in females, from early resilience to later vulnerability, highlights the need to identify underlying compensatory mechanisms and the shifting points that drive disease progression.

Future research must integrate sex as a fundamental biological variable and focus on longitudinal, mechanistic studies linking molecular, cellular, and functional changes. Particular attention should be given to microglial phenotypic transitions, neuroinflammatory processes, and emerging synaptic biomarkers of pre-symptomatic stage (*i.e.*, synaptosomal-associated protein-25 (SNAP-25) and synaptotagmin-1 (SYP-1) (Villa et al., 2020). Understanding these sex-specific trajectories will be essential for developing targeted and more effective therapeutic strategies, and for clarifying the potential, yet still unresolved, role of hormone-based interventions in modifying AD risk and progression (Song et al., 2020).

## References

[B1] Alberdi E. Sánchez-Gómez M. V. Cavaliere F. Pérez-Samartín A. Zugaza J. L. Trullas R. et al. (2010). Amyloid β oligomers induce Ca2+ dysregulation and neuronal death through activation of ionotropic glutamate receptors. *Cell Calcium* 47 264–272. 10.1016/j.ceca.2009.12.010 20061018

[B2] Amatniek J. C. Hauser W. A. DelCastillo-Castaneda C. Jacobs D. M. Marder K. Bell K. et al. (2006). Incidence and predictors of seizures in patients with Alzheimer’s disease. *Epilepsia* 47 867–872. 10.1111/j.1528-1167.2006.00554.x 16686651

[B3] Aranda-Abreu G. E. Rojas-Durán F. Hernández-Aguilar M. E. Herrera-Covarrubias D. Tlapa-Monge L. R. Mestizo-Gutiérrez S. L. (2026). Alzheimer’s disease as a disorder of neuroimmune dysregulation. *Neurol. Int.* 18:37. 10.3390/neurolint18020037 41745721 PMC12943405

[B4] Auta J. Locci A. Guidotti A. Davis J. M. Dong H. (2022). Sex-dependent sensitivity to positive allosteric modulation of GABA action in an APP knock-in mouse model of Alzheimer’s disease: Potential epigenetic regulation. *Curr. Res. Neurobiol.* 3:100025. 10.1016/j.crneur.2021.100025 36518344 PMC9743067

[B5] Barko K. Shelton M. Xue X. Afriyie-Agyemang Y. Puig S. Freyberg Z. et al. (2022). Brain region- and sex-specific transcriptional profiles of microglia. *Front. Psychiatry* 13:945548. 10.3389/fpsyt.2022.945548 36090351 PMC9448907

[B6] Bonham L. W. Sirkis D. W. Yokoyama J. S. (2019). The transcriptional landscape of microglial genes in aging and neurodegenerative disease. *Front. Immunol.* 10:1170. 10.3389/fimmu.2019.01170 31214167 PMC6557985

[B7] Carello-Collar G. Bellaver B. Ferreira P. C. L. Ferrari-Souza J. P. Ramos V. G. Therriault J. et al. (2023). The GABAergic system in Alzheimer’s disease: A systematic review with meta-analysis. *Mol. Psychiatry* 28 5025–5036. 10.1038/s41380-023-02140-w 37419974

[B8] Chaigneau T. Sha S. Roux C. M. Aïd S. Faucher A. Chantran Y. et al. (2025). Subtle alterations in hippocampal neuronal activity coincide with early sex-specific differences in amyloidosis and microglia in a pre-symptomatic mouse model of alzheimer-like pathology. *Glia* 73 1727–1745. 10.1002/glia.70029 40304030

[B9] Copenhaver A. E. LeGates T. A. (2024). Sex-specific mechanisms underlie long-term potentiation at hippocampus-nucleus accumbens synapses. *bioRxiv [Preprint]* 10.1101/2024.01.15.575709 38806250 PMC11223474

[B10] Counts S. E. Che S. Ginsberg S. D. Mufson E. J. (2011). Gender differences in neurotrophin and glutamate receptor expression in cholinergic nucleus basalis neurons during the progression of Alzheimer’s disease. *J. Chem. Neuroanat.* 42 111–117. 10.1016/j.jchemneu.2011.02.004 21397006 PMC3155625

[B11] Demarest T. G. McCarthy M. M. (2015). Sex differences in mitochondrial (dys)function: Implications for neuroprotection. *J. Bioenerget. Biomembranes* 47 173–188. 10.1007/s10863-014-9583-7 25293493 PMC4988325

[B12] Dennison J. L. Ricciardi N. R. Lohse I. Volmar C.-H. Wahlestedt C. (2021). Sexual dimorphism in the 3xTg-AD mouse model and its impact on pre-clinical research. *J. Alzheimer’s Dis.* 80 41–52. 10.3233/JAD-201014 33459720 PMC8075398

[B13] Edwards L. La Joie R. Iaccarino L. Strom A. Baker S. L. Casaletto K. B. et al. (2021). Multimodal neuroimaging of sex differences in cognitively impaired patients on the Alzheimer’s continuum: Greater tau-PET retention in females. *Neurobiol. Aging* 105 86–98. 10.1016/j.neurobiolaging.2021.04.003 34049062 PMC8820163

[B14] Ethiraj J. Palpagama T. H. Turner C. Van Der Werf B. Waldvogel H. J. Faull R. L. M. et al. (2021). The effect of age and sex on the expression of GABA signaling components in the human hippocampus and entorhinal cortex. *Sci. Rep.* 11:21470. 10.1038/s41598-021-00792-8 34728681 PMC8563768

[B15] Ferretti M. T. Cavedo E. Chiesa P. A. Schumacher Dimech A. Santuccione Chadha A. Baracchi F. et al. (2018). Sex differences in Alzheimer disease—The gateway to precision medicine. *Nat. Rev. Neurol.* 14 457–469. 10.1038/s41582-018-0032-9 29985474

[B16] Fortel I. Zhan L. Ajilore O. Wu Y. Mackin S. Leow A. (2023). Disrupted excitation-inhibition balance in cognitively normal individuals at risk of Alzheimer’s disease. *J. Alzheimer’s Dis.* 95 1449–1467. 10.3233/JAD-230035 37718795 PMC11260287

[B17] Foy M. R. Xu J. Xie X. Brinton R. D. Thompson R. F. Berger T. W. (1999). 17β-estradiol enhances NMDA receptor-mediated EPSPs and long-term potentiation. *J. Neurophysiol.* 81 925–929. 10.1152/jn.1999.81.2.925 10036289

[B18] Gleichmann M. Chow V. W. Mattson M. P. (2011). Homeostatic disinhibition in the aging brain and Alzheimer’s disease. *J. Alzheimer’s Dis.* 24 15–24. 10.3233/JAD-2010-101674 21187584 PMC4543386

[B19] Granger M. W. Franko B. Taylor M. W. Messier C. George-Hyslop P. Bennett S. A. L. (2016). A TgCRND8 mouse model of Alzheimer’s disease exhibits sexual dimorphisms in behavioral indices of cognitive reserve. *J. Alzheimer’s Dis.* 51 757–773. 10.3233/JAD-150587 26890738

[B20] Guillot-Sestier M.-V. Araiz A. R. Mela V. Gaban A. S. O’Neill E. Joshi L. et al. (2021). Microglial metabolism is a pivotal factor in sexual dimorphism in Alzheimer’s disease. *Commun. Biol.* 4:711. 10.1038/s42003-021-02259-y 34112929 PMC8192523

[B21] He P. Zhong Z. Lindholm K. Berning L. Lee W. Lemere C. et al. (2007). Deletion of tumor necrosis factor death receptor inhibits amyloid β generation and prevents learning and memory deficits in Alzheimer’s mice. *J. Cell Biol.* 178 829–841. 10.1083/jcb.200705042 17724122 PMC2064547

[B22] Irwin R. W. Yao J. To J. Hamilton R. T. Cadenas E. Brinton R. D. (2012). Selective oestrogen receptor modulators differentially potentiate brain mitochondrial function. *J. Neuroendocrinol.* 24 236–248. 10.1111/j.1365-2826.2011.02251.x 22070562 PMC3264398

[B23] Jankowsky J. L. Zheng H. (2017). Practical considerations for choosing a mouse model of Alzheimer’s disease. *Mol. Neurodegeneration* 12:89. 10.1186/s13024-017-0231-7 29273078 PMC5741956

[B24] Jiang H. He P. Xie J. Staufenbiel M. Li R. Shen Y. (2014). Genetic deletion of TNFRII gene enhances the Alzheimer-like pathology in an APP transgenic mouse model via reduction of phosphorylated I B. *Hum. Mol. Genet.* 23 4906–4918. 10.1093/hmg/ddu206 24824215 PMC4148612

[B25] Jiao S.-S. Bu X.-L. Liu Y.-H. Zhu C. Wang Q.-H. Shen L.-L. et al. (2016). Sex dimorphism profile of Alzheimer’s disease-type pathologies in an APP/PS1 mouse model. *Neurotoxicity Res.* 29 256–266. 10.1007/s12640-015-9589-x 26707129

[B26] Jiménez-Herrera R. Contreras A. Djebari S. Mulero-Franco J. Iborra-Lázaro G. Jeremic D. et al. (2023). Systematic characterization of a non-transgenic Aβ1-42 amyloidosis model: Synaptic plasticity and memory deficits in female and male mice. *Biol. Sex Dif.* 14: 59. 10.1186/s13293-023-00545-4 37716988 PMC10504764

[B27] Kang S. S. Ebbert M. T. W. Baker K. E. Cook C. Wang X. Sens J. P. et al. (2018). Microglial translational profiling reveals a convergent APOE pathway from aging, amyloid, and tau. *J. Exp. Med.* 215 2235–2245. 10.1084/jem.20180653 30082275 PMC6122978

[B28] Kang S. Kim J. Lee S.-Y. Okamura N. Chang K.-A. (2022). MicroPET imaging assessment of brain tau and amyloid deposition in 6 × Tg Alzheimer’s disease model mice. *Int. J. Mol. Sci.* 23:5485. 10.3390/ijms23105485 35628296 PMC9146140

[B29] Keren-Shaul H. Spinrad A. Weiner A. Matcovitch-Natan O. Dvir-Szternfeld R. Ulland T. K. et al. (2017). A unique microglia type associated with restricting development of Alzheimer’s disease. *Cell* 169 1276–1290.e17. 10.1016/j.cell.2017.05.018 28602351

[B30] Klinge C. M. (2017). Estrogens regulate life and death in mitochondria. *J. Bioenerget. Biomembranes* 49 307–324. 10.1007/s10863-017-9704-1 28401437

[B31] Krasemann S. Madore C. Cialic R. Baufeld C. Calcagno N. El Fatimy R. et al. (2017). The TREM2-APOE pathway drives the transcriptional phenotype of dysfunctional microglia in neurodegenerative diseases. *Immunity* 47 566–581.e9. 10.1016/j.immuni.2017.08.008 28930663 PMC5719893

[B32] Lecordier S. Pons V. Rivest S. ElAli A. (2022). Multifocal cerebral microinfarcts modulate early Alzheimer’s Disease pathology in a sex-dependent manner. *Front. Immunol.* 12:813536. 10.3389/fimmu.2021.813536 35173711 PMC8841345

[B33] Lenz K. M. Nugent B. M. Haliyur R. McCarthy M. M. (2013). Microglia are essential to masculinization of brain and behavior. *J. Neurosci.* 33 2761–2772. 10.1523/JNEUROSCI.1268-12.2013 23407936 PMC3727162

[B34] Li H. Xie Z. Tian Y. Zhou R. Yang Y. Lin B. et al. (2026). Genome-wide consensus transcriptional signatures identify synaptic pruning linking Alzheimer’s disease and epilepsy. *Mol. Psychiatry* 31 1774–1784. 10.1038/s41380-025-03318-0 41139712

[B35] Logan S. M. Sarkar S. N. Zhang Z. Simpkins J. W. (2011). Estrogen-induced signaling attenuates soluble Aβ peptide-mediated dysfunction of pathways in synaptic plasticity. *Brain Res.* 1383 1–12. 10.1016/j.brainres.2011.01.038 21262203 PMC3065978

[B36] Lopez-Lee C. Torres E. R. S. Carling G. Gan L. (2024). Mechanisms of sex differences in Alzheimer’s disease. *Neuron* 112 1208–1221. 10.1016/j.neuron.2024.01.024 38402606 PMC11076015

[B37] Lynch M. A. (2024). A case for seeking sex-specific treatments in Alzheimer’s disease. *Front. Aging Neurosci.* 16:1346621. 10.3389/fnagi.2024.1346621 38414633 PMC10897030

[B38] Mango D. Saidi A. Cisale G. Y. Feligioni M. Corbo M. Nisticò R. (2019). Targeting synaptic plasticity in experimental models of Alzheimer’s disease. *Front. Pharmacol.* 10:778. 10.3389/fphar.2019.00778 31379566 PMC6646937

[B39] Mathys H. Davila-Velderrain J. Peng Z. Gao F. Mohammadi S. Young J. Z. et al. (2019). Single-cell transcriptomic analysis of Alzheimer’s disease. *Nature* 570 332–337. 10.1038/s41586-019-1195-2 31042697 PMC6865822

[B40] Meda L. Cassatella M. A. Szendrei G. I. Otvos L. Baron P. Villalba M. et al. (1995). Activation of microglial cells by β-amyloid protein and interferon-γ. *Nature* 374 647–650. 10.1038/374647a0 7715705

[B41] Mi Y. Qi G. Brinton R. D. Yin F. (2021). Mitochondria-targeted therapeutics for Alzheimer’s disease: The good, the bad, the potential. *Antioxidants Redox Signaling* 34 611–630. 10.1089/ars.2020.8070 32143551 PMC7891225

[B42] Mifflin M. A. Winslow W. Surendra L. Tallino S. Vural A. Velazquez R. (2021). Sex differences in the IntelliCage and the Morris water maze in the APP/PS1 mouse model of amyloidosis. *Neurobiol. Aging* 101 130–140. 10.1016/j.neurobiolaging.2021.01.018 33610962 PMC8122060

[B43] Mitrovic A. D. Maddison J. E. Johnston G. A. R. (1999). Influence of the oestrous cycle on L-glutamate and l-aspartate transport in rat brain synaptosomes. *Neurochem. Int.* 34 101–108. 10.1016/S0197-0186(98)00066-7 10213067

[B44] Navakkode S. Gaunt J. R. Pavon M. V. Bansal V. A. Abraham R. P. Chong Y. S. et al. (2021). Sex-specific accelerated decay in time/activity-dependent plasticity and associative memory in an animal model of Alzheimer’s disease. *Aging Cell* 20:e13502. 10.1111/acel.13502 34796608 PMC8672784

[B45] Nilsen L. H. Melø T. M. Witter M. P. Sonnewald U. (2014). Early differences in dorsal hippocampal metabolite levels in males but not females in a transgenic rat model of Alzheimer’s disease. *Neurochem. Res.* 39 305–312. 10.1007/s11064-013-1222-x 24338370

[B46] Nong Y. Kim J. S. Jia L. Arancio O. Wang Q. (2025). The interaction between neurotransmitter receptor activity and amyloid-β pathology in Alzheimer’s disease. *J. Alzheimer’s Dis.* 106 391–409. 10.1177/13872877251342273 40388923 PMC12283230

[B47] Olmos G. Lladó J. (2014). Tumor necrosis factor alpha: A link between neuroinflammation and excitotoxicity. *Mediators Inflammation* 2014 1–12. 10.1155/2014/861231 24966471 PMC4055424

[B48] Oveisgharan S. Arvanitakis Z. Yu L. Farfel J. Schneider J. A. Bennett D. A. (2018). Sex differences in Alzheimer’s disease and common neuropathologies of aging. *Acta Neuropathol.* 136 887–900. 10.1007/s00401-018-1920-1 30334074 PMC6279593

[B49] Palomero-Gallagher N. Bidmon H. Zilles K. (2003). AMPA, kainate, and NMDA receptor densities in the hippocampus of untreated male rats and females in estrus and diestrus. *J. Comp. Neurol.* 459 468–474. 10.1002/cne.10638 12687711

[B50] Palop J. J. Mucke L. (2010). Amyloid-β–induced neuronal dysfunction in Alzheimer’s disease: From synapses toward neural networks. *Nature Neuroscience* 13 812–818. 10.1038/nn.2583 20581818 PMC3072750

[B51] Pluta R. (2025). Sex-specific adaptations in Alzheimer’s disease and ischemic stroke: A longitudinal study in male and female APPswe/PS1dE9 mice. *Life* 15:1146. 10.3390/life15071146 40141679 PMC11944048

[B52] Pluta R. Ułamek-Kozioł M. (2026). Genomic and proteomic conversion of brain ischemia to Alzheimer’s disease. *Front. Cell Dev. Biol.* 14:1804251. 10.3389/fcell.2026.1804251 42222332 PMC13216197

[B53] Podcasy J. L. Epperson C. N. (2016). Considering sex and gender in Alzheimer disease and other dementias. *Dialogues Clin. Neurosci.* 18 437–446. 10.31887/DCNS.2016.18.4/cepperson 28179815 PMC5286729

[B54] Riedel B. C. Thompson P. M. Brinton R. D. (2016). Age, APOE and sex: Triad of risk of Alzheimer’s disease. *J. Steroid Biochem. Mol. Biol.* 160 134–147. 10.1016/j.jsbmb.2016.03.012 26969397 PMC4905558

[B55] Rissman R. A. Mobley W. C. (2011). Implications for treatment: GABAA receptors in aging, down syndrome and Alzheimer’s disease: GABA receptors in age and disease. *J. Neurochem.* 117 613–622. 10.1111/j.1471-4159.2011.07237.x 21388375 PMC3127285

[B56] Rodrigues K. D. C. Oliveira M. D. C. De Souza I. C. Igansi A. W. Lopes A. V. B. De Oliveira Pacheco C. et al. (2025). Sex-dependent therapeutic effects of nano-curcumin on alzheimer’s disease: Enhanced cognitive and physiological restoration in female mice. *Psychopharmacology* 10.1007/s00213-025-06967-9 [Epub ahead of print]. 41284069

[B57] Roveta F. Bonino L. Piella E. M. Rainero I. Rubino E. (2024). Neuroinflammatory biomarkers in Alzheimer’s disease: From pathophysiology to clinical implications. *Int. J. Mol. Sci.* 25:11941. 10.3390/ijms252211941 39596011 PMC11593837

[B58] Roy U. Stute L. Höfling C. Hartlage-Rübsamen M. Matysik J. Roβner S. et al. (2018). Sex- and age-specific modulation of brain GABA levels in a mouse model of Alzheimer’s disease. *Neurobiol. Aging* 62 168–179. 10.1016/j.neurobiolaging.2017.10.015 29154037

[B59] Rueda-Carrasco J. Sokolova D. Lee S. Childs T. Jurèáková N. Crowley G. et al. (2023). Microglia-synapse engulfment via PtdSer-TREM2 ameliorates neuronal hyperactivity in Alzheimer’s disease models. *EMBO J.* 42:e113246. 10.15252/embj.2022113246 37575021 PMC10548173

[B60] Sala Frigerio C. Wolfs L. Fattorelli N. Thrupp N. Voytyuk I. Schmidt I. et al. (2019). The major risk factors for Alzheimer’s disease: Age, sex, and genes modulate the microglia response to Aβ plaques. *Cell Rep.* 27 1293–1306.e6. 10.1016/j.celrep.2019.03.099 31018141 PMC7340153

[B61] Sawai T. Bernier F. Fukushima T. Hashimoto T. Ogura H. Nishizawa Y. (2002). Estrogen induces a rapid increase of calcium-calmodulin-dependent protein kinase II activity in the hippocampus. *Brain Res.* 950 308–311. 10.1016/S0006-8993(02)03186-4 12231258

[B62] Schwarz J. M. Sholar P. W. Bilbo S. D. (2012). Sex differences in microglial colonization of the developing rat brain. *J. Neurochem.* 120 948–963. 10.1111/j.1471-4159.2011.07630.x 22182318 PMC3296888

[B63] Sha S. Chaigneau T. Krantic S. (2023). Pre-symptomatic synaptic dysfunction and longitudinal decay of hippocampal synaptic function in APPPS1 mouse model of Alzheimer’s disease is sex-independent. *Brain Res. Bull.* 198 36–49. 10.1016/j.brainresbull.2023.04.005 37080395

[B64] Song Y. Li S. Li X. Chen X. Wei Z. Liu Q. et al. (2020). The effect of estrogen replacement therapy on Alzheimer’s disease and Parkinson’s disease in postmenopausal women: A meta-analysis. *Front. Neurosci.* 14:157. 10.3389/fnins.2020.00157 32210745 PMC7076111

[B65] Soni N. D. Swain A. Juul H. Cao Q. Haris M. Wolk D. A. et al. (2024). Detection of sex-specific glutamate changes in subregions of hippocampus in an early-stage Alzheimer’s disease mouse model using GluCEST MRI. *Alzheimer’s Dement.* 20 7124–7137. 10.1002/alz.14190 39262197 PMC11485308

[B66] Steeland S. Libert C. Vandenbroucke R. E. (2018). A new venue of TNF targeting. *Int. J. Mol. Sci.* 19:1442. 10.3390/ijms19051442 29751683 PMC5983675

[B67] Stephen T. L. Cacciottolo M. Balu D. Morgan T. E. LaDu M. J. Finch C. E. et al. (2019). APOE genotype and sex affect microglial interactions with plaques in Alzheimer’s disease mice. *Acta Neuropathol. Commun.* 7:82. 10.1186/s40478-019-0729-z 31113487 PMC6528326

[B68] Strefeler A. Jan M. Quadroni M. Teav T. Rosenberg N. Chatton J.-Y. et al. (2023). Molecular insights into sex-specific metabolic alterations in Alzheimer’s mouse brain using multi-omics approach. *Alzheimer’s Res. Therapy* 15:8. 10.1186/s13195-023-01162-4 36624525 PMC9827669

[B69] Tabatadze N. Huang G. May R. M. Jain A. Woolley C. S. (2015). Sex differences in molecular signaling at inhibitory synapses in the hippocampus. *J. Neurosci.* 35 11252–11265. 10.1523/JNEUROSCI.1067-15.2015 26269634 PMC4532757

[B70] Tenkorang M. A. Snyder B. Cunningham R. L. (2018). Sex-related differences in oxidative stress and neurodegeneration. *Steroids* 133 21–27. 10.1016/j.steroids.2017.12.010 29274405 PMC5864532

[B71] Villa A. Gelosa P. Castiglioni L. Cimino M. Rizzi N. Pepe G. et al. (2018). Sex-specific features of microglia from adult mice. *Cell Rep.* 23 3501–3511. 10.1016/j.celrep.2018.05.048 29924994 PMC6024879

[B72] Villa C. Lavitrano M. Salvatore E. Combi R. (2020). Molecular and imaging biomarkers in Alzheimer’s disease: A focus on recent insights. *J. Pers. Med.* 10:61. 10.3390/jpm10030061 32664352 PMC7565667

[B73] Wang S. Wang S. Chen H. Xu J. (2026). Microglia–neuron crosstalk: An intimate molecular conversation in neurodegeneration. *Int. J. Mol. Sci.* 27:2011. 10.3390/ijms27042011 41752147 PMC12940662

[B74] Wang Y. Ma Y. Hu J. Cheng W. Jiang H. Zhang X. et al. (2015). Prenatal chronic mild stress induces depression-like behavior and sex-specific changes in regional glutamate receptor expression patterns in adult rats. *Neuroscience* 301 363–374. 10.1016/j.neuroscience.2015.06.008 26071959

[B75] Wang Y. Wang J. Chen X. Lin Z. You Z. He K. et al. (2025). Tau pathology is associated with postsynaptic metabotropic glutamate receptor 5 (mGluR5) in early Alzheimer’s disease in a sex-specific manner. *Alzheimer’s Dement.* 21:e70004. 10.1002/alz.70004 39998900 PMC11853735

[B76] Zhang A. Y. Elias E. White A. G. Jones L. G. Hamad T. Manners M. T. (2025). Microglia reactivity is brain region and sex specific in the context of chronic stress. *Sci. Rep.* 15:33285. 10.1038/s41598-025-18000-2 41006452 PMC12475140

[B77] Zhao W. Hou Y. Zhang Q. Yu H. Meng M. Zhang H. et al. (2023). Estrogen receptor β exerts neuroprotective effects by fine-tuning mitochondrial homeostasis through NRF1/PGC-1α. *Neurochem. Int.* 171:105636. 10.1016/j.neuint.2023.105636 39491237

[B78] Zlotnik A. Gruenbaum B. F. Mohar B. Kuts R. Gruenbaum S. E. Ohayon S. et al. (2011). The effects of estrogen and progesterone on blood glutamate levels: Evidence from changes of blood glutamate levels during the menstrual cycle in women. *Biol. Reproduction* 84 581–586. 10.1095/biolreprod.110.088120 20980684

